# Immunization coverage and timeliness of vaccination in young patients with inborn errors of metabolism: a French multicentric study

**DOI:** 10.1186/s13023-025-03648-w

**Published:** 2025-03-31

**Authors:** Anne-Sophie Renous, Lena Damaj, Magali Gorce, Magalie Barth, Antoine Bedu, Elise Sacaze, Delphine Lamireau, Cécile Laroche-Raynaud, Laurent Pasquier, Zoha Maakaroun-Vermesse, Marine Tardieu, François Labarthe

**Affiliations:** 1 Centre de référence Maladies Métaboliques ToTeM, Service de Médecine Pédiatrique, Hôpital Clocheville, CHRU Tours, 49 Bd Béranger, 37 044 Tours Cedex 1, Tours, France; 2 Competence Center for Inborn Errors of Metabolism, CHU Rennes, Rennes, France; 3 Reference Center for Inborn Errors of Metabolism, CHU Toulouse, Toulouse, France; 4 Department of Medical Genetics, CHU Angers, Angers, France; 5 Competence Center for Inborn Errors of Metabolism, CHU Limoges, Limoges, France; 6 Competence Center for Inborn Errors of Metabolism, CHU Brest, Brest, France; 7 Competence Center for Inborn Errors of Metabolism, CHU Bordeaux, Bordeaux, France; 8 Vaccination Center, CHU Tours, Tours, France; 9 INSERM U1069, Nutrition, Croissance et Cancer, Université de Tours, Tours, France

**Keywords:** Vaccine, Prevention, Vaccination delay, Vaccination schedule, Vulnerable patient

## Abstract

**Background:**

Inborn errors of metabolism (IEMs) are rare disorders that are heterogeneous in severity and clinical presentation. Patients with IEMs should receive the vaccination schedule recommended for the whole population, and specific vaccinations, such as the seasonal influenza vaccine, for the most vulnerable. The aim of this study was to evaluate vaccination coverage and timeliness in young patients with an IEM.

**Patients & methods:**

We conducted a retrospective multicentric (7 centers) study between February 2021 and May 2022 evaluating vaccination coverage and delays in French young patients with an IEM according to the yearly French vaccination schedules published since 2002. The results were analyzed considering patient health conditions as stable or at risk (defined as cardiorespiratory failure or by an IEM with a serious risk of metabolic crisis).

**Results:**

Two hundred seventy-five patients were enrolled in this study. Among them, only 164 (60%) were up-to-date with the standard French vaccination schedule, and 229 (83%) had received at least one vaccine from this schedule late. The rate of delayed vaccination was significantly greater in the at-risk group than in the stable group for the main primaries and first booster doses of the DTaP-IPV-Hib vaccine and for the first MMR injection. Finally, only 30 to 35% of at-risk patients were vaccinated against influenza during the three previous winters.

**Conclusion:**

Young patients with an IEM had insufficient vaccination coverage with significant delays, exposing them to vaccine-preventable diseases, particularly at-risk patients with cardiorespiratory failure or a serious risk of metabolic crisis. Furthermore, only a few of the most vulnerable patients had received specific vaccinations, such as the influenza vaccine. Therefore, optimizing vaccination within the recommended schedule is crucial for this population of vulnerable children who have regular hospital follow-up.

**Supplementary Information:**

The online version contains supplementary material available at 10.1186/s13023-025-03648-w.

## Introduction

Inborn errors of metabolism (IEMs) are rare disorders caused by genetic deficiencies of an enzyme or transporter. The clinical presentation and severity of these diseases vary widely. Individually, each IEM is rare, but its overall prevalence is estimated to be approximately 1 per 2,000 live births [1]. Some (mostly deficits in amino acid catabolism and certain abnormalities in energy metabolism) are at risk of acute metabolic crisis, especially in catabolic states. Others, such as lysosomal storage diseases, lead to multisystem degradation with progressive cardiac or respiratory failure. These children are therefore particularly vulnerable to infectious diseases and must be protected by vaccination. A recent review of literature summarizes the safety and recommendations for vaccinations in patients with IEMs, focusing on the risk of decompensation after any degree of metabolic stress associated to vaccine preventable diseases [2]. However, several studies have shown vaccination gaps within the population of children with IEMs. In 2011, a multicenter study conducted in California reported lower vaccination coverage among children classified as having stable IEM than among control subjects [3]. A monocentric French study dating from 2015 confirmed lower vaccination coverage among children followed for IEM than among a control group [4].

Beyond vaccination coverage, the concept of vaccination delay is also an important indicator, reflecting the quality of vaccination during vulnerable ages for vaccine-preventable diseases. Delaying the first vaccine doses in infants increases the period of vulnerability to these diseases. For example, pertussis outbreaks occur regularly, with particularly high morbidity and mortality rates among young infants [5]. Definitions of potentially harmful vaccination delays have been established by expert opinions via the Delphi process [6]. A multicenter study conducted in France in 2014 reported vaccination delays in 47% of children attending primary care [7]. Several studies have assessed vaccination delays in children with chronic diseases [8, 9], but none have specifically focused on patients with IEMs.

The primary objective of this multicenter study was to evaluate vaccination coverage and delays in a population of young patients with IEMs. The secondary objectives were to assess vaccination coverage and delay according to the stability of the disease (stable IEM versus a high risk of metabolic crisis or cardiorespiratory failure), compare vaccination coverage to national data, and describe vaccination coverage for specifically indicated vaccines not included in the standard vaccination schedule, such as influenza vaccination.

## Patients and methods

### Patients

We conducted a retrospective multicentric study of vaccination coverage and delay in young patients with IEMs. Patients were enrolled in 7 French centers for IEMs (Angers, Brest, Bordeaux, Limoges, Rennes, Toulouse, and Tours) between February 2021 and May 2022. The inclusion criteria were an age between 6 months and 20 years, a diagnosis of IEM for at least 6 months, a follow-up at a specialized center for IEM, and consultation at that center during the inclusion period. Patients without available vaccination data (unavailable health records), those vaccinated according to a non-French vaccination schedule, or those with a contraindication to vaccination were excluded.

### Procedure and data collection

The data collected for each patient included age, type of IEM and date of diagnosis, potential risk of acute metabolic decompensation, presence of respiratory and/or heart failure, and finally, the presence of a contraindication to a vaccine and any adverse event considered serious and associated with a previous vaccination. The vaccination data were collected from the patients’ health records and included the type of vaccine and injection date. All the data were anonymized at each center and transferred via email via a secure messaging system.

### Definitions, vaccination schedule and delay

IEMs were classified according to their classical pathophysiology into IEMs related to small-molecule disorders (accumulation or deficiency), complex-molecule disorders (accumulation, deficiency, or cell processing and trafficking defects) and energy defects (membrane transporters and cytosolic and mitochondrial energy defects) [10]. Patients were separated into two groups according to their health condition: stable or at-risk. At-risk conditions were defined by cardiorespiratory failure or by an IEM with a serious risk of metabolic crisis. The analysis of vaccination data considered the yearly French vaccination schedules published since 2002 (available at the site of the French Ministry of Health, https://sante.gouv.fr/), accounting for the evolution of vaccination recommendations. The timeframe corresponding to detrimental vaccination delay was defined according to the study by Gras et al. [6]. This study, which uses a 3-round Delphi process, obtained an expert consensus to define a potentially dangerous vaccination delay for each dose of each vaccine recommended for children younger than 2 years of age, according to the 2015 French immunization schedule. These definitions are summarized in Table 1. For other vaccinations (schedules before 2015 and subsequent modifications), vaccination delay was empirically defined as a timeframe relative to the recommended injection date, exceeding 15 days for injections recommended in the first 6 months of life, exceeding 2 months for injections from 6 months to 6 years, and exceeding 1 year for vaccines after the age of 6 years. The delay durations presented in the results corresponded to the timeframe beyond the detrimental delay. When a vaccine had not yet been administered, the child was considered unvaccinated. We also compared the vaccination coverage of our group of patients with an IEM to that of the French population at the age of 2 years. National vaccination coverage data at 24 months were extrapolated from national surveys by Santé Publique France (available on the site https://www.santepubliquefrance.fr/), weighted for each vaccine by the number of patients in the corresponding birth year.

**Table 1:** Proposed definition of the potentially damaging vaccination delay (example according to the French 2015 vaccination schedule)

Vaccination dose	Current recommended age for routine immunization (French 2015 schedule)	Potentially damaging vaccination delay
DTaP-IPV	2 months	> 15 days
	4 months	> 15 days
	11 months	> 2 months
	6 years	> 1 year
	11–13 years	> 1 year
Hib	2 months	> 15 days
	4 months	> 15 days
	11 months	> 2 months
PCV	2 months	> 15 days
	4 months	> 15 days
	11 months	> 2 months
MMR	12 months	> 1 month
	16–18 months	> 6 months
MenC	12 months	> 1 month
VHB	2 months	> 11 years
	4 months	> 11 years
	11 months	> 11 years

Vaccination delay was defined according to Gras et al. [6] or extrapolated for booster doses after 2 years of age. DTaP-IPV: diphtheria, tetanus, acellular pertussis-inactivated poliomyelitis vaccine; Hib: Haemophilus influenzae b vaccine; PCV: pneumococcal conjugate vaccine; MenC meningococcal C vaccine; MMR: measles mumps rubella vaccine. HBV hepatitis B vaccine

### Ethics approval and consent to participate

A written information letter was provided to legal representatives, and non-opposition to participation in the study was obtained. Patients or their parents who refused to participate were not included. No nominative, sensitive or personal data were collected from the patients. This study was registered locally at our institution and was approved by our local ethics committee (n°2024–018).

### Statistical analysis

The results were expressed as the median [min‒max], mean ± SD or number of subjects (%), as appropriate. The quantitative data were compared via the Mann‒Whitney test. Qualitative data were compared via Fisher’s exact test. All the statistical analyses were carried out via GraphPad Prism version 6.0 (GraphPad software, Inc.). A p value < 0.05 was considered to indicate statistical significance.

## Results

### Patients

**Fig. 1:**
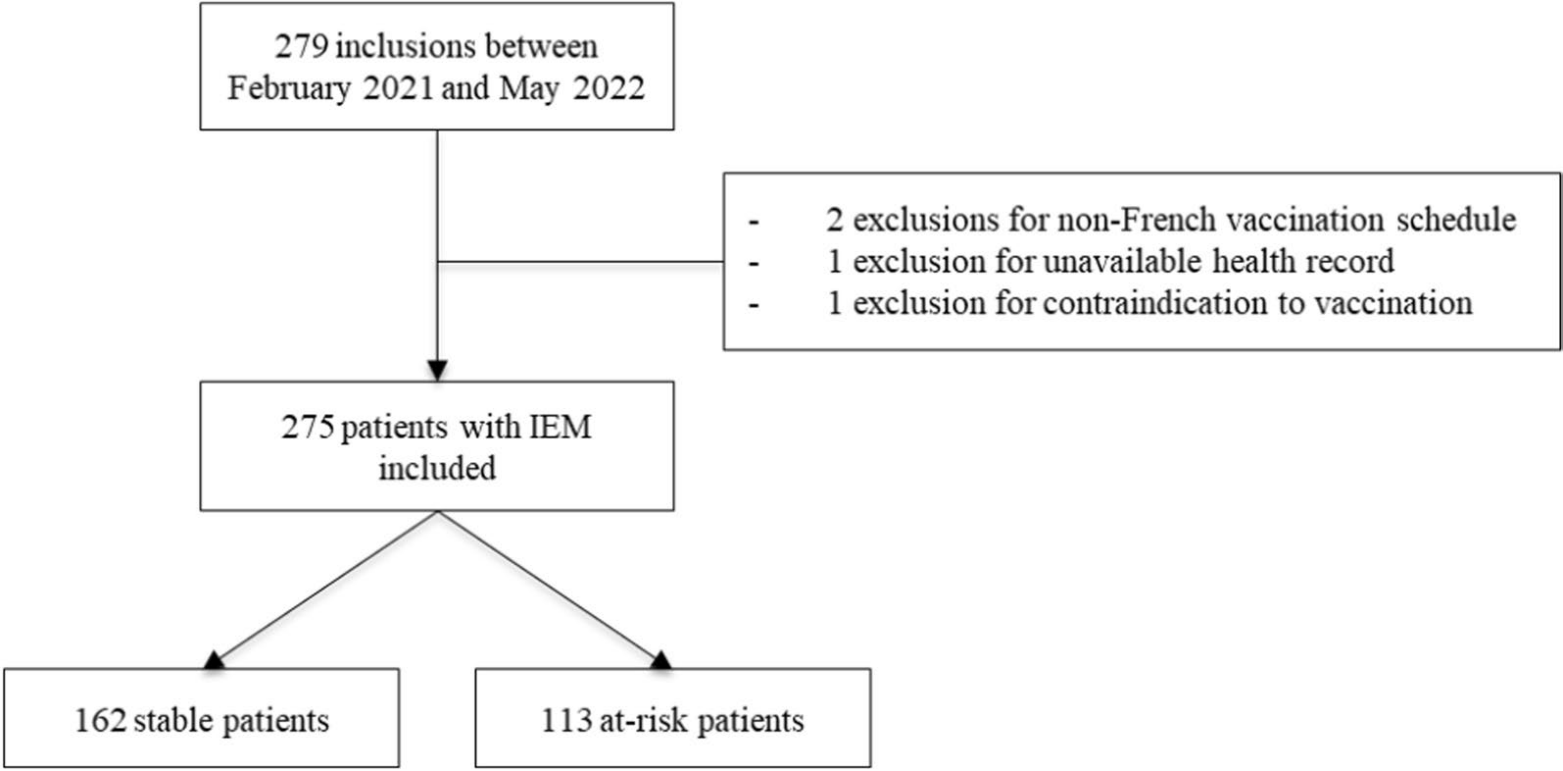
Flow chart

Two hundred seventy-nine patients were enrolled in this study (see Flow chart Fig. 1) between February 2021 and May 2022; 4 were excluded because of a non-French vaccination schedule (n = 2), unavailable health records (n = 1), or contraindications to vaccination related to recent hematopoietic stem cell transplantation (n = 1). Finally, 275 patients were analyzed and divided into stable (n = 162) and at-risk (n = 113) patients. The main characteristics of the patients are reported in Table 2. Briefly, the age of the patients at inclusion varied between 6 months and 19.8 years, with a median age of 8 years, whereas the median age at diagnosis was 0.6 months. A majority of patients had an IEM related to a defect in small molecule metabolism (64%), whereas disorders of complex molecules and energy defects represented 17% and 19% of the patients, respectively. Compared with at-risk patients, stable patients were significantly younger at diagnosis and had a greater proportion of patients with small molecule disorders, which was probably related to the greater number of patients with phenylketonuria (n = 94). In contrast, complex molecule disorders were more common in at-risk patients (33% *vs* 6%, *p* < 0.001). Age at inclusion was not different between the two groups.

**Table 2:** Patient characteristics

	All patients (n = 275)	Stable patients (n = 162)	At-risk patients (n = 113)	*p value*
Age at inclusion (months)	95 [6–237]	93 [7–237]	101 [6–225]	*NS*
Age at diagnosis (months)	0.6 [0.0–180.6]	0.3 [0.0–180.6]	7.7 [0.0–164.4]	< *0.001*
Group of IEM:				
Small molecule disorders	177 (64%)	119 (73%)	58 (51%)	< *0.001*
Complex molecule disorders	47 (17%)	9 (6%)	38 (33%)	< *0.001*
Energy defects	51 (19%)	34 (21%)	17 (15%)	< *0.001*

The results are presented as the median [min–max] or number of patients (%). p value: Mann‒Whitney test or Fisher’s exact test as appropriate; stable versus at-risk patients; NS: nonsignificant (*p* ≥ 0.05). IEM: inborn errors of metabolism

### Vaccination coverage and delays for all patients

No serious adverse events associated with vaccination were reported. The results of vaccination coverage are presented in Table 3. Briefly, immunization against diphtheria, tetanus, acellular pertussis, inactivated poliomyelitis and *Haemophilus influenzae b* (DTaP-IPV and Hib) was satisfactory, with primary vaccination coverage ranging between 98 and 100%. The first booster dose was almost systematically administered (vaccination coverage between 96 and 100%), whereas the next booster doses were given to 87% to 95% of the patients. The immunization against pneumococcus was also satisfactory, with primary vaccination coverage ranging between 91 and 99%, and a booster dose was given to 92% of the patients. Ninety-seven% of the patients had received 2 doses of the measles-mumps-rubella (MMR) vaccine. Most of the patients (91%) received 3 doses of the hepatitis B vaccine. As expected, the vaccination coverage for the Meningococcal C vaccine was lower (84% for the first dose and 74% for the second dose). Finally, a complete vaccination schedule was used for only 60% of the patients. Taken together, these results were unsatisfactory given the insufficient vaccination coverage of patients with IEMs.

**Table 3:** Vaccination coverage and delay of 275 patients with inborn errors of metabolism

	Vaccination coverage	Vaccination with delay	Duration of delay (months)
DT-IPV 1st dose	275 (100%)	83 (30%)	0.69 [0.03–19.07]
DT-IPV 2nd dose	125 (99%)	61 (48%)	0.81 [0.02–53.77]
DT-IPV 3rd dose	275 (100%)	130 (47%)	0.83 [0.00–55.60]
DT-IPV 1st booster	267 (100%)	68 (25%)	3.93 [0.12–66.76]
DT-IPV 2nd booster	156 (95%)	36 (22%)	6.71 [0.00–73.64]
DT-IPV 3rd booster	59 (88%)	4 (6%)	5.05 [1.84–24.53]
Hib 1st dose	273 (99%)	81 (29%)	0.62 [0.03–13.97]
Hib 2nd dose	122 (98%)	59 (47%)	0.77 [0.02–53.77]
Hib 3rd dose	273 (99%)	128 (47%)	0.81 [0.00–55.60]
Hib booster	258 (96%)	59 (22%)	3.08 [0.12–64.69]
aP 1st dose	275 (100%)	83 (30%)	0.69 [0.03–19.07]
aP 2nd dose	125 (99%)	61 (48%)	0.82 [0.02–56.60]
aP 3rd dose	275 (100%)	130 (47%)	0.83 [0.00–67.83]
aP 1st booster	264 (99%)	67 (25%)	3.87 [0.12–112.99]
aP 2nd booster	121 (87%)	23 (17%)	4.87 [0.16–54.58]
aP 3rd booster	59 (88%)	4 (6%)	5.05 [1.84–24.53]
HBV 3 doses	230 (91%)	1 (1%)	190
PCV 1st dose	262 (99%)	71 (27%)	0.75 [0.03–140.12]
PCV 2nd dose	62 (91%)	23 (34%)	0.97 [0.05–10.44]
PCV 3rd dose	254 (97%)	103 (39%)	0.76 [0.00–19.30]
PCV booster	234 (92%)	80 (31%)	3.19 [0.15–115.66]
MenC 1st dose	230 (84%)	110 (40%)	6.76 [0.02–126.25]
MenC 2nd dose	55 (74%)	24 (32%)	0.82 [0.02–66.17]
MMR 1st dose	261 (97%)	110 (41%)	1.93 [0.01–73.32]
MMR 2nd dose	248 (97%)	76 (30%)	7.65 [0.07–119.32]
Complete vaccination schedule	164 (60%)	229 (83%)	1.31 [0.00;140.11]

The results are presented as the number of patients (%) or median [min–max]. Abbreviations of the vaccine names are detailed in Table 1

Furthermore, vaccination delay was frequent for a majority of the vaccines. Almost half of the patients received their primary vaccinations for DTaP-IPV, Hib and PCV with a delay. This delay was longer than 1 month in one-third of the cases, with maximal values of approximately 4–5 years. A vaccination delay was less frequent and shorter for booster doses. A vaccination delay was also frequent for other vaccines (concerning 30 to 40% of the patients for MenC and MMR vaccines), except for the HBV vaccine (for whom vaccinations are recommended during the first year of life, but vaccination delay is defined by the absence of vaccination after the age of 11 years). Taken together, a vaccination delay was reported in 83% of the patients.

Compared with national data at the age of 2 years, the vaccination coverage of patients with an IEM was in the same range as that of the French population (Table S1 in supplementary data files), at least for the primary vaccinations, whereas it seems to be slightly lower for booster doses. Again, a vaccination delay appears to be the explanation because the coverage of these booster doses was satisfactory in the results irrespective of patient age (Table 3).

### Stable versus at-risk patients

Insufficient vaccination coverage was detected in both groups (Table S2 in the supplementary data), with no difference between stable and at-risk patients, except for the percentage of patients receiving the HBV vaccine, which was lower for at-risk patients (86% *vs* 95%, *p* < 0.05). The vaccination schedule was completed in 64% of stable patients and in 54% of at-risk patients (NS). However, a vaccination delay was significantly more frequent in at-risk patients for the primary doses and for the first booster doses of DTaP-IPV and Hib (Table 4). The difference did not reach significance for the second and third booster doses. A vaccination delay for the 1st dose of the MMR vaccine was also more frequent in at-risk patients (54% *vs* 34%, *p* < 0.01). More in details, differences existed between the various types of IEMs, with a minimal rate of vaccinations with delay in PKU patients (median 16%), contrasting with others IEMs with a maximal rate of vaccinations with delay in patients with others aminoacidopathies (Maple Syrup Urine Disease, type 1 tyrosinemia, etc., median 53%, *p* < 0.05, Table S3 in the supplementary data). The rate of delay also increased significantly with age, but this effect remained of limited importance (linear regression, *p* < 0.0001, R2 = 0.078). However, the duration of vaccination delay was mostly the same between stable and at-risk patients (Table S4 in the supplementary data).

**Table 4:** Vaccination delay of stable versus at-risk patients with inborn errors of metabolism

	Stable patients (n = 162)	At-risk patients (n = 113)	*p* value
DT-IPV 1st dose	38 (23%)	45 (40%)	*0.005*
DT-IPV 2nd dose	29 (41%)	32 (58%)	*NS*
DT-IPV 3rd dose	65 (40%)	65 (58%)	*0.005*
DT-IPV 1st booster	32 (20%)	36 (33%)	*0.022*
DT-IPV 2nd booster	19 (22%)	17 (25%)	*NS*
DT-IPV 3rd booster	1 (3%)	3 (11%)	*NS*
Hib 1st dose	38 (24%)	43 (39%)	*0.007*
Hib 2nd dose	29 (41%)	30 (58%)	*NS*
Hib 3rd dose	65 (40%)	63 (57%)	*0.009*
Hib booster	27 (18%)	32 (30%)	*0.023*
aP 1st dose	38 (23%)	45 (40%)	*0.005*
aP 2nd dose	29 (41%)	32 (58%)	*NS*
aP 3rd dose	65 (40%)	65 (58%)	*0.005*
aP 1st booster	31 (20%)	36 (33%)	*0.015*
aP 2nd booster	15(18%)	8 (18%)	*NS*
aP 3rd booster	1 (3%)	3 (12%)	*NS*
HBV 3 doses	1 (1%)	0 (0%)	*NS*
PCV 1st dose	35 (23%)	36 (34%)	*NS*
PCV 2nd dose	8 (28%)	15 (45%)	*NS*
PCV 3rd dose	53 (34%)	50 (50%)	*0.018*
PCV booster	40 (28%)	40 (43%)	*0.017*
MenC 1st dose	67 (48%)	43 (48%)	*NS*
MenC 2nd dose	16 (43%)	8 (44%)	*NS*
MMR 1st dose	52 (34%)	58 (54%)	*0.002*
MMR 2nd dose	40 (27%)	36 (35%)	*NS*
Complete vaccination schedule	134 (83%)	95 (84%)	*NS*

The results are presented as the number of patients vaccinated with delay (% of vaccinated patients). p value: Fisher’s exact test stable vs at risk; NS: nonsignificant (*p* ≥ 0.05)

### Specifically indicated vaccines

The seasonal flu vaccine was administered to a limited number of patients (approximately one-third of at-risk patients, whereas it was recommended for all these patients and less than 10% of stable patients; Table 5). Patients vaccinated with varicella vaccine were also rare (18% of at-risk patients and 3% of stable patients), but our collected data did not include the notion of a past history of chickenpox. The BCG vaccine has been used in 21% of patients, with no difference between stable and at-risk conditions. This vaccine remains recommended in France, but only for children with an identified risk factor for tuberculosis. The others optional vaccinations remained very limited in patients with IEMs.

**Table 5:** Vaccination coverage for specifically indicated vaccines of patients with inborn errors of metabolism

	All patients (n = 275)	Stable patients (n = 162)	At-risk patients (n = 113)	*p value*
Influenza 2018/2019	34 (16%)	6 (6%)	27 (30%)	< *0.0001*
Influenza 2019/2020	42 (18%)	8 (6%)	34 (35%)	< *0.0001*
Influenza 2020/2021	48 (19%)	10 (7%)	38 (35%)	< *0.0001*
HPV (2 doses)	8 (11%)	6 (15%)	2 (6%)	*NS*
BCG	59 (21%)	39 (24%)	20 (18%)	*NS*
PPV 23	8 (3%)	2 (1%)	6 (6%)	*NS*
Varicella	23 (8%)	4 (3%)	19 (18%)	< *0.0001*
Men B	7 (3%)	4 (3%)	3 (3%)	*NS*
Men ACWY	4 (1%)	1 (1%)	3 (3%)	*NS*
Rotavirus (2 doses)	12 (5%)	3 (2%)	9 (9%)	*0.014*
HAV	5 (2%)	2 (1%)	3 (3%)	*NS*

The results are presented as the number of vaccinated patients (%). p value: Fisher’s exact test stable vs at risk; NS: nonsignificant (p ≥ 0.05). BCG bacillus Calmette-Guerin; HPV human papillomavirus; PPV 23 23-valent pneumococcal polysaccharide vaccine; HAV hepatitis A virus vaccine

## Discussion

This study demonstrated insufficient immunization coverage among French patients with IEMs. Moreover, a significant number of vaccinations were performed with potentially harmful delays. The percentage of patients vaccinated with delay was greater in the at-risk group than in the stable group. Furthermore, the coverage of specifically indicated vaccines remained very limited in patients with IEMs, including seasonal flu vaccines, which were rarely used even in at-risk patients.

Vaccination is one of the most cost-effective public health interventions, as it prevents the costs associated with treating and caring for people who become ill. To achieve community immunization, the objective of the World Health Organization (WHO) Expanded Program on Immunization is to reach vaccination coverage of 90% [11]. In our study, vaccination coverage among the population of young patients with IEM exceeded 90% for the first vaccine doses. However, it fell below this threshold for later doses, including the third DT-IPV booster, the second and third pertussis vaccine boosters, and the second dose of the meningococcal C vaccine. Furthermore, for an individual benefit, and given that these patients had a chronic illness and received close medical follow-up, vaccination coverage higher than that of the whole population was expected, ideally close to 100% [12]. A majority of patients with IEMs are at increased risk of vaccine-preventable diseases and hence require additional strategies to maximize protection against these diseases [4, 13].

In our study, 83% of patients with IEMs received at least one vaccination from the vaccination schedule late. In fact, measuring standard vaccination coverage does not reflect whether children received vaccine doses at the recommended ages or whether vaccines were given concomitantly per the schedule. Quantifying vaccination timeliness, i.e., comparing when children receive vaccine doses relative to age recommendations, is also important for the success of an expanded program on immunization [14]. Vaccination delay seems to be frequent in the whole population. Two studies conducted in the United States involving 16,211 infants aged 24–35 months [15] and 9,223 children aged 25–72 months [16] reported vaccination delay rates of 58% and 48%, respectively. Similar results were also recently reported in France, with primary care pediatricians reporting that 47% (among 443 children) had at least one potentially dangerous immunization delay [7]. A timely vaccination is even more expected for patients with chronic diseases. However, a recent French study, which used the same definitions of vaccination delay as ours, highlights a vaccination delay rate of 26% to 75% (depending on the vaccine) among children with chronic diseases [7, 9]. Since children with chronic diseases represent a primary target for immunization strategies, it is important that their immunization coverage and the timeliness of vaccination are optimal. Improving timeliness and minimizing missed opportunities to vaccinate individuals with these special risk conditions will also optimize protection from vaccine-preventable diseases [12]. Our recommendation is that children with IEMs should be strictly monitored for routine and recommended vaccinations and that health care providers and families should be properly informed to avoid false contraindications (such as a risk of inducing a metabolic crisis). Thus, Klein et al*.* reported that vaccination of children with IEM (n = 271) was not associated with any significant increase in emergency-department visits or hospitalizations during the 30 days after vaccination [3]. Protection can also include ‘cocooning’ (i.e., ensuring appropriate immunizations within the immediate family; e.g., varicella, influenza and pertussis vaccination).

Compared with national data at the age of 2 years, the vaccination coverage rates for primary vaccinations (DTaP-IPV, Hib, and PCV) among patients with IEMs were globally similar to those reported for the whole population, according to data from Santé Publique France. However, for booster doses, vaccination coverage appears to be lower among patients with IEMs. Moreover, vaccination coverage for the first dose of MMR was similar, whereas for the second dose, it was lower among patients with IEM than among the whole population. Given that the overall vaccination coverage of both populations was almost similar, these results suggest that in our patients with IEM, subsequent doses are administered but delayed. Pandolfi et al*.* also reported that vaccination coverage among children with chronic diseases increased between the ages of 12 and 24 months, indicating that vaccines in the first year of life are often administered late [8]. However, these data should be interpreted prudently because we extrapolated vaccination coverage data from the general population. This methodology did not allow for statistical comparison. Using Northern California Kaiser Permanente’s electronic medical records, Klein et al*.* compared vaccination coverage data at 24 months from 77 infants with IEM to 1540 matched control subjects and reported no difference [3].

In further analyses, we found similar vaccination coverage between patients with at-risk IEM and patients with stable IEM, except for hepatitis B vaccination. In their study, Cerutti et al*.* reported some differences in vaccination coverage within a population of 132 patients with IEMs, which were analyzed in three groups (sickest, chronic, and stable). Patients defined as chronic had lower vaccination coverage for pneumococcus, and those defined as sickest had lower vaccination coverage for meningococcus [4]. There was a greater rate of vaccination delay among at-risk patients than among stable patients. These results are particularly concerning, as they indicate that the most vulnerable patients are protected with delay against vaccine-preventable diseases. Cerutti et al*.* also reported that patients with IEMs in the sickest group were vaccinated at a later age than healthy controls and had lower vaccination coverage than patients with chronic and stable IEMs [4].

Furthermore, in our study, the coverage rate for specifically indicated vaccines was low in the at-risk group of IEM patients. Indeed, 30 to 35% of these patients were vaccinated against influenza during the winters of 2018, 2019, and 2020. This rate is similar to that reported by Cerutti et al*.* in their population of patients with IEMs (35 to 50%) [4]. Diallo et al*.* reported an influenza vaccination coverage of 15% among a population of 146 patients with various chronic diseases [9]. Immunization with the 23-valent pneumococcal polysaccharide vaccine remained exceptional (6% of at-risk patients), whereas the presence of a chronic disease also predisposes children to invasive pneumococcal diseases, with a high mortality rate that has been reported to be 25 times higher among children with chronic disease [17]. Finally, it was not possible to determine the lower rate of patients vaccinated with the varicella vaccine because we did not include the notion of a past history of chickenpox. Overall, our study revealed inadequate vaccine coverage for specifically indicated vaccines among young patients with IEMs.

Our study was not designed to investigate the reasons of this insufficient vaccination coverage with significant delays, but we can formulate some hypotheses on the reasons for this vaccination delay. Factors contributing to vaccination delays have been largely reported in the whole population [18], and a “Catalogue of interventions addressing vaccine hesitancy” is available on the website of the European Centre for Disease Prevention and Control (ECDC) and proposed various tools for helping healthcare workers. Additional factors specific to our group of patients also exist [2]. First, there may be parental or medical fear of exacerbating chronic IEM, which is linked to potential adverse events of vaccines, such as fever, increased energy demands or a catabolic state [3, 19]. Cases of metabolic crisis following vaccination have been described in the literature, revealing IEMs [20, 21]. However, when the IEM is well known, vaccination is not associated with serious adverse events [3, 22]. The safety of vaccines in patients with IEMs has been largely demonstrated in recent literature, including those with live attenuated viruses [2]. The fear from metabolic crises has been refuted, even in high-risk groups, such as urea cycle disorders or mitochondrial diseases. Some experts still recommend special precautions and careful monitoring after vaccine administration to patients with IEMs prone to rapid metabolic decompensation or immunodeficiency [19]. In any case, vaccination prevention in children with IEMs must therefore be ensured according to the vaccination schedule, such as healthy children in the whole population and those receiving the COVID-19 vaccine [23]. The only exception is the rotavirus vaccine (containing a source of fructose), which is contraindicated for hereditary fructose intolerance [24]. Individuals with IEMs need to be up-to-date with their immunizations. These children may also warrant specific vaccination indications, such as seasonal influenza vaccination or enhanced pneumococcal vaccination, for example, in cases of respiratory failure [23, 25]. Vaccinations may also be delayed due to an intercurrent event related or unrelated to the chronic illness. For vaccinations not recommended for the whole population, such as influenza or expanded pneumococcal vaccination, there may be a lack of awareness, both parental and medical, of eligibility for these vaccines. Finally, these children are often followed up by several doctors (pediatricians and specialists), and the responsibility for updating the vaccination schedule is sometimes not assured, with one doctor assuming that another will do it. Additionally, as in the general population, parental vaccine refusal due to vaccine hesitancy may exist, particularly in France, where declining confidence and acceptance in vaccines and immunization programs have been recently reported [26]. These different causes are identified in the literature for children in the whole population, such as those with IEMs [4, 7].

We recognize that our study had several limitations. First, we did not have a control group from the whole population. However, the use of national data from Santé Publique France provides a rough estimate of differences with the whole population. Another limitation is that we did not collect data on the patients’ sex, which hinders the interpretation of HPV vaccination. Finally, the design of our study did not allow us to evaluate the causes of vaccination absence or delays.

**Fig. 2:**
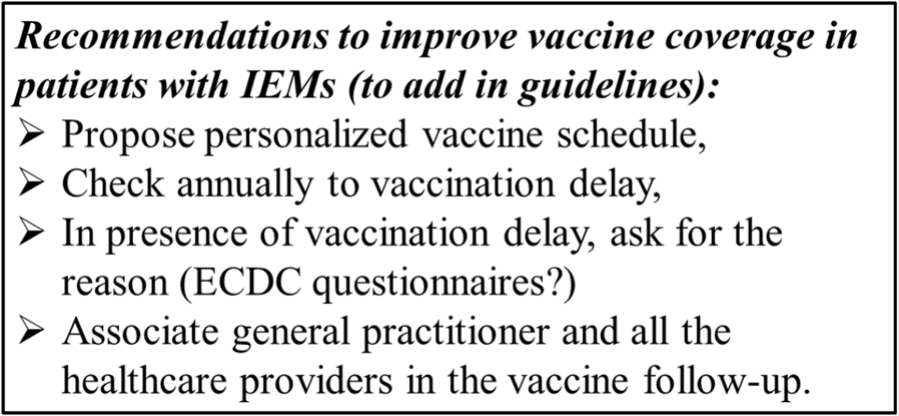
Recommendations to improve vaccine coverage in patients with IEMs (to add in guidelines)

Many patients with IEMs are especially vulnerable if unprotected against vaccine preventable diseases (intoxication or energy metabolism disorders, presence of cardiac and/or respiratory failure, etc.). Moreover, several IEMs are associated with some level of immunodeficiency (ex: congenital disorder of glycosylation, glycogen storage disease type Ib), which can require a personalized vaccine schedule. National or international guidelines exist for the most frequent IEMs. A first suggestion to improve vaccination coverage in these patients is to include in guidelines recommendations for vaccine schedule, including if necessary personalized vaccine schedule. A second suggestion is to include in the annual visit to check for vaccination delay. In presence of vaccination delay, the reason for this delay must be questioned, with the possibility to use the ECDC questionnaires, and resolved. Finally, the general practitioner and all the healthcare providers involved in the patient’s care must be associated in the recommendations to vaccinate the patient (Fig. 2).

## Conclusion

The present study demonstrated inadequate vaccine coverage and significant delays among young patients with IEMs, especially at-risk patients with cardiorespiratory failure or with a serious risk of metabolic crisis. It is essential to communicate with parents and healthcare providers involved in the follow-up of patients with IEMs about the importance of administering vaccinations at the ages recommended by the vaccination schedule. Indeed, achieving timely vaccination coverage against vaccine-preventable diseases is crucial, especially among children with at-risk IEMs. These conditions can indeed decompensate during infections, leading to a catabolic state. It can also increase the vulnerability of these patients to infection complications. Finally, it is also necessary to optimize vaccinations with specific recommendations, such as annual influenza or varicella vaccination, for patients with particular vulnerabilities among those with IEMs.

## Supplementary Information

Supplementary material 1

## Data Availability

The datasets used and analyzed during the current study are available from the corresponding author upon reasonable request.

## Abbreviations

aP: Acellular pertussis vaccine
BCG: Bacillus Calmette–Guérin
DT-IPV: Diphtheria, tetanus, inactivated poliomyelitis vaccine
HAV and HBV: Hepatitis A and B virus vaccines
Hib: *Haemophilus influenzae* Type B vaccine
HPV: Human papillomavirus vaccine
IEM: Inborn errors of metabolism
Men ACWY and B: Meningococcal ACWY and B vaccines
MMR: Measles-mumps-rubella vaccine
NS: Nonsignificant
PCV: Pneumococcal conjugate vaccine
PPV 23: 23-Valent pneumococcal polysaccharide vaccine
SD: Standard deviation
