# Supplementary material for: Immunization coverage and timeliness of vaccination in young patients with inborn errors of metabolism: a French multicentric study
Source: Orphanet J Rare Dis. 2025 Mar 31;20:149. doi: 10.1186/s13023-025-03648-w (PMC11959845; doi:10.1186/s13023-025-03648-w)
Supplement: Supplementary file 1 — Supplementary material 1 [file 13023_2025_3648_MOESM1_ESM.docx]

**Supplementary data**

**Table S1: Vaccination coverage of young patients with inborn errors of metabolism at the age of 24 months compared with national vaccination coverage**

|  | Patients with IEM (n=275) | French vaccination coverage (1) |
| --- | --- | --- |
| DT-IPV primary vaccinations | 241 (99%) | 99% |
| DT-IPV 1^st^ booster | 217 (89%) | 93% |
|  |  |  |
| Hib primary vaccinations | 239 (98%) | 97% |
| Hib booster* | 209 (89%) | 91% |
|  |  |  |
| aP primary vaccinations | 241 (99%) | 98% |
| aP 1^st^ booster | 216 (89%) | 92% |
|  |  |  |
| HBV (3 doses) | 177 (77%) | 70% |
|  |  |  |
| PCV (3 doses)** | 151 (90%) | 91% |
|  |  |  |
| MenC*** | 94 (63%) | 66% |
|  |  |  |
| MMR 1^st^ dose** | 173 (93%) | 90% |
| MMR 2^nd^ dose** | 115 (61%) | 76% |

*The results are presented as the number of vaccinated patients (%). (1) Data for French vaccination coverage are extrapolated from surveys conducted by Santé Publique France (*[*https://www.santepubliquefrance.fr/*](https://www.santepubliquefrance.fr/)*) considering the year of birth of our patients (patients born since *2004, **2010 and ***2011).*

**Table S2: Vaccination coverage of stable versus at-risk patients with inborn errors of metabolism**

|  | Stable patients  (n=162) | At-risk patients  (n=113) | *p value* |
| --- | --- | --- | --- |
| DT-IPV 1^st^ dose | 162 (100%) | 113 (100%) | *NS* |
| DT-IPV 2^nd^ dose | 70 (100%) | 55 (98%) | *NS* |
| DT-IPV 3^rd^ dose | 162 (100%) | 113 (100%) | *NS* |
| DT-IPV 1^st^ booster | 158 (100%) | 109 (99%) | *NS* |
| DT-IPV 2^nd^ booster | 88 (95%) | 68 (94%) | *NS* |
| DT-IPV 3^rd^ booster | 32 (89%) | 27 (87%) | *NS* |
|  |  |  |  |
| Hib 1^st^ dose | 162 (100%) | 111 (98%) | *NS* |
| Hib 2^nd^ dose | 70 (100%) | 52 (95%) | *NS* |
| Hib 3^rd^ dose | 162 (100%) | 111 (98%) | *NS* |
| Hib booster | 153 (97%) | 105 (95%) | *NS* |
|  |  |  |  |
| aP 1^st^ dose | 162 (100%) | 113 (100%) | *NS* |
| aP 2^nd^ dose | 70 (100%) | 55 (98%) | *NS* |
| aP 3^rd^ dose | 162 (100%) | 113 (100%) | *NS* |
| aP 1^st^ booster | 156 (99%) | 108 (98%) | *NS* |
| aP 2^nd^ booster | 77 (92%) | 44 (80%) | *NS* |
| aP 3^rd^ booster | 32 (91%) | 27 (84%) | *NS* |
|  |  |  |  |
| HBV 3 doses | 139 (95%) | 91 (86%) | *0.012* |
|  |  |  |  |
| PCV 1^st^ dose | 155 (99%) | 107 (98%) | *NS* |
| PCV 2^nd^ dose | 29(85%) | 33 (97%) | *NS* |
| PCV 3^rd^ dose | 154(99%) | 100 (94%) | *NS* |
| PCV booster | 142 (93%) | 92 (90%) | *NS* |
|  |  |  |  |
| MenC 1st dose | 140 (86%) | 90 (80%) | *NS* |
| MenC 2^nd^ dose | 37 (74%) | 18 (75%) | *NS* |
|  |  |  |  |
| MMR 1^st^ dose | 153 (96%) | 108 (98%) | *NS* |
| MMR 2^nd^ dose | 146 (99%) | 102 (95%) | *NS* |
|  |  |  |  |
| Complete vaccination schedule | 103 (64%) | 61 (54%) | *NS* |

*The results are presented as the number of vaccinated patients (%). p value: Chi-square test stable vs. at-risk; NS: nonsignificant (p≥0.05).*

**Table S3: Rate of vaccination delay according to the type of IEM**

|  | n | Rate of vaccination with delay (%) | *p value* |
| --- | --- | --- | --- |
| Phenylketonuria (PKU) | 94 | 16% [0-87%] | *-* |
| Other aminoacidopathies | 14 | 53% [12-84%] | *P<0.001* |
| Organic acidemias | 19 | 33% [0-83%] | *NS* |
| Urea Cycle Disorders | 35 | 41% [0-74%] | *NS* |
| Galactosemias | 15 | 27% [0-70%] | *NS* |
| Fatty acid oxidation disorders | 20 | 30% [0-83%] | *NS* |
| Glycogen Storage Diseases | 12 | 29% [0-70%] | *NS* |
| Mitochondrial cytopathies | 15 | 27% [0-88%] | *NS* |
| Lysosomal Storage Disorders | 17 | 33% [0-85%] | *NS* |
| Other complex-molecule disorders | 34 | 19% [0-71%] | *NS* |

*The results are presented as the number of patients, median rate of vaccination delay (% [min-max]). One-way ANOVA confirmed differences between type of IEM (p=0.005) with Bonferroni's post-Test comparing each type of IEM with PKU (p value), NS: nonsignificant.*

**Table S4: Duration of vaccination delay in stable versus at-risk patients with inborn errors of metabolism**

|  | Stable patients  (n=162) | At-risk patients  (n=113) | *p value* |
| --- | --- | --- | --- |
| DT-IPV 1^st^ dose | 0.69 [0.03-11.60] | 0.69 [0.03-19.07] | *NS* |
| DT-IPV 2^nd^ dose | 0.71 [0.02-11.59] | 0.92 [0.05-53.77] | *NS* |
| DT-IPV 3^rd^ dose | 0.60 [0.00-11.97] | 1.02 [0.00-55.60] | *0.029* |
| DT-IPV 1^st^ booster | 3.85 [0.15-53.51] | 4.26 [0.12-66.76] | *NS* |
| DT-IPV 2^nd^ booster | 4.87 [0.00-65.42] | 9.53 [0.30-73.64] | *NS* |
| DT-IPV 3^rd^ booster | 1.84 | 5.19 [4.90-24.53] | *--* |
|  |  |  |  |
| Hib 1^st^ dose | 0.69 [0.03-13.97] | 0.62 [0.03-5.59] | *NS* |
| Hib 2^nd^ dose | 0.69 [0.02-12.58] | 0.84 [0.05-53.77] | *NS* |
| Hib 3^rd^ dose | 0.60 [0.00-23.54] | 0.99 [0.00-55.60] | *NS* |
| Hib booster | 3.28 [0.15-25.73] | 3.05 [0.12-64.69] | *NS* |
|  |  |  |  |
| aP 1^st^ dose | 0.69 [0.03-11.60] | 0.69 [0.03-19.07] | *NS* |
| aP 2^nd^ dose | 0.74 [0.02-11.59] | 0.97 [0.05-56.60] | *NS* |
| aP 3^rd^ dose | 0.60 [0.00-11.97] | 1.12 [0.00-67.83] | *0.020* |
| aP 1^st^ booster | 3.70 [0.15-112.99] | 4.26 [0.12-66.76] | *NS* |
| aP 2^nd^ booster | 3.68 [0.16-12.85] | 8.17 [0.30-54.58] | *NS* |
| aP 3^rd^ booster | 1.84 | 5.19 [4.90-24.53] | *--* |
|  |  |  |  |
| HBV 3 doses | 190 | -- | *--* |
|  |  |  |  |
| PCV 1^st^ dose | 0.85 [0.03-24.20] | 0.69 [0.03-140.12] | *NS* |
| PCV 2^nd^ dose | 1.05 [0.05-3.11] | 0.97 [0.12-10.44] | *NS* |
| PCV 3^rd^ dose | 0.79 [0.00-19.30] | 0.63 [0.00-15.82] | *NS* |
| PCV booster | 3.55 [0.15-23.51] | 3.09 [0.23-115.66] | *NS* |
|  |  |  |  |
| MenC 1st dose | 5.74 [0.02-126.25] | 9.03 [0.19-97.25] | *NS* |
| MenC 2^nd^ dose | 0.71 [0.02-66.17] | 1.32 [0.22-9.49] | *NS* |
|  |  |  |  |
| MMR 1^st^ dose | 1.47 [0.01-33.57] | 2.57 [0.02-73.32] | *0.045* |
| MMR 2^nd^ dose | 5.28 [0.17-106.33] | 11.66 [0.07-119.32] | *NS* |
|  |  |  |  |
| Complete vaccination schedule | 1.35 [0.00-126.25] | 1.29 [0.00-140.12] | *NS* |

*The results are presented as the median duration of vaccination delay in months [min–max]. p value: Mann‒Whitney test, stable vs. at-risk; NS: nonsignificant (p≥0.05)*
